# Adapting Artificial Crowns to Roots of Teeth

**Published:** 1884-06

**Authors:** Geo. W. Smith


### Adapting Artificial Crowns to Roots of Teeth.

BY DR. GEO. W. SMITH.

Read Before the Mississippi Valley Dental Society.

The practice of adapting artificial crowns to the roots of decayed teeth is the first method in which artificial work was introduced to the dental profession. And to-day there is no phase of the mechanical department which promises greater success than the one we are about to discuss.

When we take into account the wideness of this subject, what it involves, and the possibilities that lie just beyond, I will scarcely be able to give you an outline in a paper like this. This method, I believe, if once brought to perfection, will give better satisfaction to the patient than any mechanical work yet discovered.

If we retrospect and acquaint ourselves with the early methods of pivoting teeth, we find that two kinds of pivots were employed, to-wit: wood and gold.

With some little alteration, the latter serves us at the present time, and doubtless will continue to do so; for we have no promise of good results when this gold, or platinum wire, is not employed, as it not only serves to hold the crown in place, but also adds strength and stability, a point of great importance.

It is not our purpose to underrate other modes of the mechanical art when we say that the signs of the times indicate that the profession is looking around for methods to replace lost dentures, other than that of inserting plates, or sacrificing part of the tissues, and destroying contour without the least hope of replacing it except by artificial means.

The fact is well known to every dentist that, with many persons, the thought of wearing an artificial plate is absolutely repulsive. This class of patients would be willing to pay almost any fee if their teeth could be restored without a plate or the removal of the wrecks remaining in the oral cavity.

The failure in treating and filling successfully the natural root was the greatest obstacle to be overcome in the adoption of a method which avoided plate work. But the rapid strides made by our progressive and ever advancing profession have at last overcome these obstacles, and by the improvements made we may now restore broken down crowns by setting gold bands, and gold and porcelain crowns on roots almost entirely destroyed by caries. Roots that are looked upon as unwelcome guests in the mouth, and that once would have been considered useless, may now be made not only to serve for keeping the muscles of the face in place, but also as good and efficient masticators.

In the erection of a grand and stately edifice, the first and most important matter to engage the attention of the architect is the foundation upon which the superstructure is to be built. So in mounting these porcelain crowns, the root, our foundation, must first be examined to see whether or not it is sound and healthy. It should be treated until it is free from all obnoxious and fermenting gases.

The pulp cavity may then be enlarged with suitable instruments, to within one-third of the apex, to receive the pivot. It has been my practice in the crown which I have originated, to cut the root short to the gum, if not slightly under the free margin on the labial surface, while on the palatine I leave it projecting slightly, if possible. I then fill the upper third of the root with Fosseline and surgeon's cotton. I take a small portion of the fibre, tease it on glass, mix it with the cement, and with small nerve instruments, I carry it to the apical foramen, completely closing it. The cotton serves to carry the cement to the desired point, and when packed in the root, will set perfectly hard, and completely exclude any egress from the canal through the apex, which is the ultimatum in all root filling.

I then take a cast from the mouth, followed by articulation, after which I select a plain plate tooth, with shade to suit those with which it is to be associated, and grind to suit the prepared root. I now select the pivot of gold or platinum, the end of which I flatten, punch holes to suit the pins in the artificial crown, and press the end on as backing. I invest this, and solder the wire to the pins. If the wire is not quite shaped to suit the hole in the root it can easily be adjusted.

Place the pivot and crown in position, and regulate the contour and articulation. Now take sheet tin, press around the palatine surface, while the tooth is in position; let the tin cover the root, also the palatine surface of the tooth. This will give the outline or pattern, from which the gold band may be cut and shaped to the root and crown; secure the band with "wax resin" and remove altogether. Invest and solder. When polished, the root, together with the crown, should be sponged off with alcohol, the mouth kept dry with bibulous paper or a napkin, until the cement is prepared for the pivot.

Mix enough of the Fosseline to fill the root, which should be put on the pivot and band, and then carry them up in position and hold firmly until it hardens. This crown is not only artistic, but serviceable; and when set, gives entire satisfaction, of which many testimonials can be had in our city at the present time.

Within a few years past, we have had a crown presented to the profession by Dr. Richmond, made of gold. This crown has found favor with many operators as a substitute for broken down molars. It meets a demand that cannot be supplied successfully, as yet, by any porcelain work for molar teeth. And I may say that many teeth have been made useful by this method that otherwise would have been lost.

The original method of setting this crown, I believe, was to dress the root, or that portion of the tooth remaining, so the crown would fit tightly when pressed to the margin of the gum. To do this it was necessary to dress the stump on which the crown was to rest, then take a cast, followed by articulation, after which we press tin foil around the stump to get the circumferance of the remaining tooth from which to make the band. The gold band may be made to fit the stump accurately by passing wire around the band, and passing the band and wire over the root on which the crown will finally rest, and with the pliers twist the wire till it fits tightly; then remove and solder, and again return to the root. Now build up with wax, and have the patient close the mouth, which will give the space to be filled with gold.

The easiest and best method in making the top is to melt the gold down on thin platinum, and after soldering to the band it may be shaped with a file to suit the articulation. An opening should be left in the top for the escape of the surplus cement, which may finally be filled with gold foil.

The dental profession can boast of many different artificial tops and varieties of porcelain crowns, all artistic, and many that may be relied on for utility and service. And we are under lasting obligations to those who are interesting themselves so energetically in behalf of the profession. But the crown that should be adopted is the one that embraces the combined elements of *strength* and *beauty*—strength to resist the pressure to which it is usually exposed, and beauty, to make it take as far as possible the place of the natural teeth.

I have now given you the results of my experience, and the method which has given me the greatest satisfaction; so that it is in order for you to relate your experiences and enter into the discussion of their merits.

Boyle, the eminent English chemist, said that if every artist would but discover what new observations occurred to *him* in the exercise of his trade, philosophy would thence gain innumerable improvements. It is just so with our profession. All knowledge began from experience, therefore all new experience is the beginning of new knowledge.

Each can accomplish much for himself by energy, hard work, and close application to his profession, but not all. So closely are we related to each other, and so different are our professional experiences that the greatest results are attained only by our meeting together, comparing notes and experiences, and discussing thoroughly the subjects that are of vital importance to our profession. Thus will the great excellencies of each one, in any particular line, be made the excellencies of all, which is a duty we owe, not only to ourselves, but to our patients. In this light there is no policy so poor as selfishness. He who seeks exclusively his own interest will never find them, for they lie not in the path he is pursuing.

When the interest of each, and the interest of all of us, are no more separated in thought and deed than they are in reality, then will the truest, surest, most permanent progress be made in our most worthy profession.

